# Identifying the Tautomeric Form of a Deoxyguanosine-Estrogen Quinone Intermediate

**DOI:** 10.3390/metabo5030475

**Published:** 2015-09-10

**Authors:** Douglas E. Stack

**Affiliations:** Department of Chemistry, University of Nebraska at Omaha, 6001 Dodge Street, Omaha, NE 68182, USA; E-Mail: dstack@unomaha.edu; Tel.: +1-402-554-3647

**Keywords:** estrogen *o*-quinones, DNA damage, carcinogenesis, apurinic sites, biomarkers

## Abstract

Mechanistic insights into the reaction of an estrogen *o*-quinone with deoxyguanosine has been further investigated using high level density functional calculations in addition to the use of 4-hyroxycatecholestrone (4-OHE_1_) regioselectivity labeled with deuterium at the C1-position. Calculations using the M06-2X functional with large basis sets indicate the tautomeric form of an estrogen-DNA adduct present when glycosidic bonds cleavage occurs is comprised of an aromatic A ring structure. This tautomeric form was further verified by use of deuterium labelling of the catechol precursor use to form the estrogen *o*-quinone. Regioselective deuterium labelling at the C1-position of the estrogen A ring allows discrimination between two tautomeric forms of a reaction intermediate either of which could be present during glycosidic bond cleavage. HPLC-MS analysis indicates a reactive intermediate with a *m*/*z* of 552.22 consistent with a tautomeric form containing no deuterium. This intermediate is consistent with a reaction mechanism that involves: (1) proton assisted Michael addition; (2) re-aromatization of the estrogen A ring; and (3) glycosidic bond cleavage to form the known estrogen-DNA adduct, 4-OHE_1_-1-N7Gua.

## 1. Introduction

**Figure 1:**
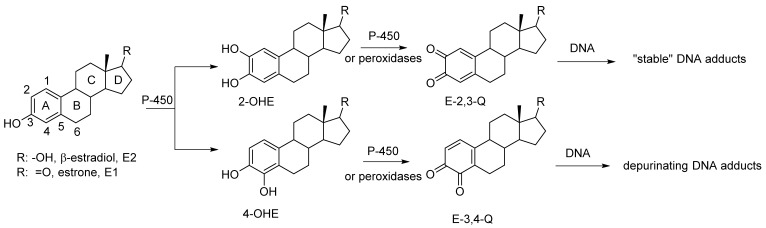
Oxidative metabolism of estrogen to catechol estrogens and estrogen *o*-quinones and subsequent reaction with DNA.

An increased level of endogenous estrogens is linked to increased rates of breast, ovarian, endometrial and other cancers [1,2,3]. The metabolism of estrogen can produced reactive metabolites capable of binding to nucleophile sites in the DNA [4]. Estrone and β-estradiol are hydroxylated by various isoforms of cytochrome P-450 [5]. This A ring hydroxylation occurs mostly at the 2-position to produce 2-hydroxycatechol estrogens (2-OHE) (Figure 1). Certain P-450 enzymes, for example P-4501B1, hydroxylate primarily at the 4-position to produced 4-hydroxycatechol estrogens (4-OHE) [6]. These catechol estrogens can acts as procarcinogens since they are readily further oxidized to estrogen *o*-quinones (EQ) [7]. The two isomeric estrogen *o*-quinones, estrogen-2,3-quinone (E-2,3-Q) and estrogen-3,4-quinone (E-3,4-Q), are both strong electrophiles but they show significant differences with their reaction to DNA [8,9].

E-3,4-Q is the more reactive electrophile and reacts primarily at the N7-position of guanine and the N3-position of adenine. Reaction at the N7-position of guanine or the N3-position of adenine results in a cationic intermediate that effects cleavage of the glycosidic bond that attaches the base to the DNA polymer. This results in an apurinic site in the DNA which must be repair with fidelity else mutations in the DNA may form.

The less reactive E-2,3-Q electrophile tautomerizes to a quinone methide and reacts the 6-position with the exocyclic amino group of either guanine or adenine [4]. This does not result in glycosidic bond cleavage and these adducts are referred to as “stable adducts”. When equal amounts of E-3,4-Q is mixed with E-2,3-Q and reacted *in vitro* with DNA, ten times more of the depurinated adducts are formed (from E-3,4-Q) when compared to stable adducts (from E-2,3-Q) [9].

Other evidence suggest that the more genotoxic route to estrogen oxidation involves formation of 4-OHE followed by further oxidation to E-3,4-Q metabolite. 4-OHE is carcinogenic in animal models prone to estrogen induced cancers whereas 2-OHE is not [10,11,12]. Increase levels of the 4-OHE hydroxylation product, as opposed to 2-OHE, has been detected in tissue in or near breast tumors [13,14]. Both increased levels of 4-OHE and the DNA adducts that result when E-3,4-Q reacts with DNA are observed in women with breast cancer and in women with high risk for breast cancer as measured by the Gail Model [15].

**Figure 2:**
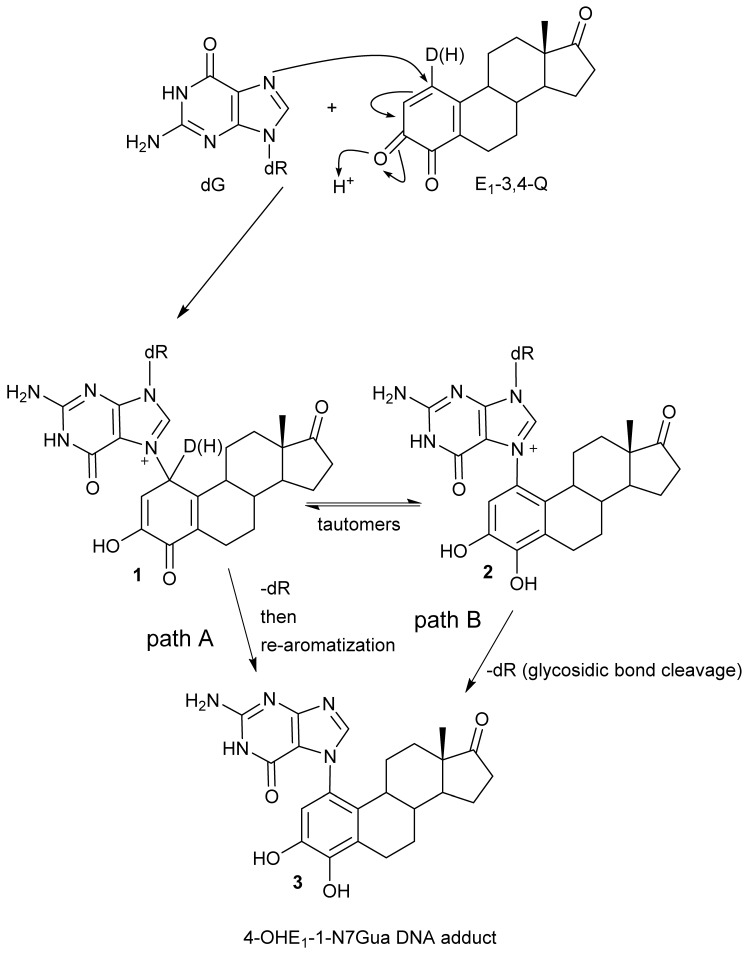
Possible mechanistic routes in the reaction of E_1_-3,4-Q (both deuterium labeled and unlabeled) with dG to form the estrogen-DNA adduct 4-OHE_1_-1-N7Gua.

We have use the *in vitro* reaction of E-3,4-Q with deoxyguanosine (dG) as a platform to investigate the mechanism and intermediates form during the production of estrogen-DNA adducts. Earlier worked demonstrated that reaction proceed through an intermediate that decomposes in a unimolecular fashion to the final 4-OHE-1-N7Gua depurinated adduct (Figure 2) [16]. It was shown that a proton assisted Michael addition leads to an intermediate that still contains the ribose moiety. This intermediate then converts to the final product by glycosidic bond cleavage.

Since the identification of this labile intermediate containing the ribose moiety was done by HPLC-MS, identification based on the parent *m*/*z* value (molecular mass) could not determine whether the intermediate structure corresponded to structure **1** or structure **2** (Figure 2) since they are tautomers and have the same molecular mass. Identification of the tautomeric form is of value to our research since we plan molecular modeling calculation into the glyosidic bond cleavage of estrogen-DNA adducts. Specifically we want to investigate the significant rate difference observed between glycosidic bond cleavage in the estrogen-adenine depurinating adduct (very fast) *versus* the slower rate of glycosidic bond cleavage in the estrogen-guanine depurinating adduct. Knowing which tautomer exists when the glycosidic bond breaks is necessary in choosing the correct structure to model.

Our prior work used UV/V is spectroscopy to determine the kinetic properties associated with the conversion of the unknown reaction intermediate to the final 4-OHE-1-N7Gua product. The conversion displayed first-order kinetics with a *t*_½_ of 40 min and a free energy of activation of 26.8 kcal/mol (ΔH^╪^ = 24.7 kcal/mol and ΔS^╪^ = 7.2 eu) [16]. Tautomer **1** could conceivably convert to the final product by rapid glycosidic bond cleavage followed by re-aromatization (tautomerization) of the estrogen A ring. If the rate of glycosidic bond cleavage was significantly faster than re-aromatization, this route (path A, Figure 2) would display the first order kinetics observed in the conversion to the final product. If re-aromatization occurs first, then glycosidic bond cleavage occurs from tautomer **2** (path B, Figure 2) and this route would also display first order kinetics.

Our original studies used a combination of molecular modeling using density functional theory, spectroscopy and assay of liberated ribose to gain mechanistic insight into the formation of 4-OHE_1_-1-N7Gua. The molecular modeling studies used abbreviated estrogen ring systems modeled with the B3LYP hybrid functional and limited basis sets. Recently, we have developed an efficient method to selectively label catechol estrogens at the unactivated 1-position of the estrogen A ring [17]. A deuterium label at this position can distinguish between structures **1** and **2** (Figure 2) with MS since tautomer **2** will be one mass unit less than tautomer **1**. The results of this labeling experiment in addition to improved modeling of both the thermodynamics and kinetics of the tautomerization of **1** to **2** are presented below.

## 2. Results and Discussion

### 2.1. Thermodynamic Calculations on the Relative Stability of Tautomers **1** and **2**

Previous quantum mechanical modeling studies indicate a significant difference in stability between tautomeric forms **1** and **2** Model compounds containing only the A and B estrogen ring system showed an energy difference of 14.6 kcal/mol at the B3LYP/6-31 + G(2d,p)//B3LYP/6-31G(d) theory with the aromatic tautomer **2** compromising the more stable form. Solvation effects were accounted for by a single-point-energy (SPE) calculation (B3LYP/6-31 + G(2d,p)) using a polarizable continuum model (PCM) and the resulting free energy of solvation was added to the gas phase geometries [16].

Substantial improvements in density functional theory, in addition to computational resources, have occurred since these initial modeling studies with an ever expanding choice of functionals developed over the past decade. Benchmarking studies of new and previous functionals has shown that the popular B3LYP functional is lacking when modeling certain molecular properties, specifically thermodynamic stabilities and reaction energies [18]. Thus we have re-examined the energy differences between tautomers **1** and **2** with the following improvements: (1) use of the meta-generalized gradient approximation (meta-GGA) functional of Zhao and Truhlar, M06-2x [19]; (2) expanding the model systems to include the A, B and C ring systems; (3) modeling the geometry in the presence of an aqueous system; (4) use of a much larger basis set for the final SPE calculation and (5) account for the diastereoisomeric (rotameric) forms the 4-OHE_1_-1-N7Gua estrogen adduct and its upstream metabolites.

**Figure 3:**
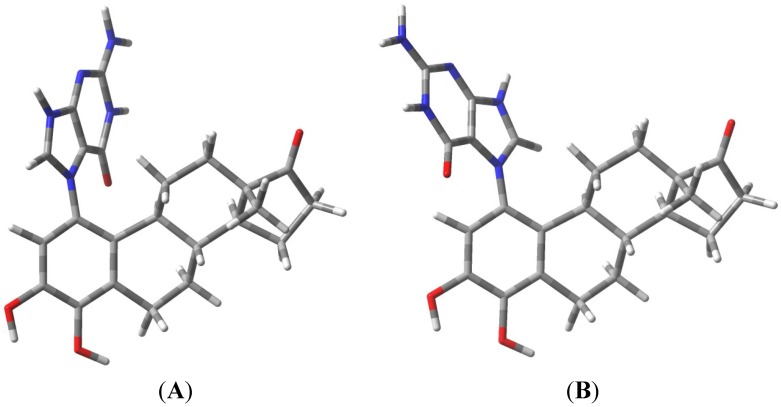
Rotameric forms of the 4-OHE_1_-1-N7Gua estrogen-DNA adduct. (**A**) The α-isomer with purine ring system pointed away from the viewer; (**B**) The β-isomer with purine ring system pointed towards the viewer.

The known, final product of the proton assisted reaction of E_1_-3,4-Q with dG is the 4-OHE_1_-1-N7Gua estrogen-DNA adduct (Figure 2). NMR characterization of the 4-OHE_1_-1-N7Gua adduct revealed the presence of two diastereoisomers caused by rotational restriction of purine moiety about the C1(estrogen)-N7(guanine) bond [4]. These rotamers were labeled as α- and β-isomers consistent with estrogen nomenclature. The β-isomer has the purine moiety on the same side of the estrogen ring system has the 17-methyl group, the α-isomer has the purine moiety directed on the other side of the ring system (Figure 3). We have modeled two sets of tautomers **1** and **2** containing the A, B and C ring systems in both the α- and β-forms using high level density functional theory with all geometry optimization and SPE done in the presence of an aqueous system, M06-2X/QZVP//M06-2X/6-31 + G(d,p) level of theory.

**Figure 4:**
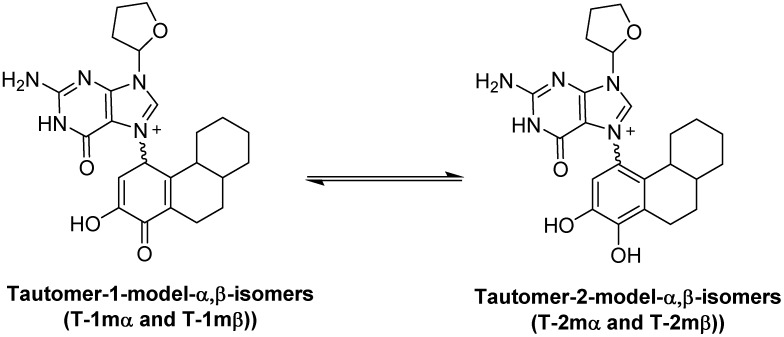
Results of M06-2X/QZVP//M06-2X/6-31 + G(d,p) calculation on model forms of tautomers **1** and **2**.

Figure 4 show the results of the density functional calculations with both the α- and β-forms of tautomers **1** and **2**. With either the α-isomer tautomers or the β-isomer tautomers, tautomeric form **2** is significantly lower in energy, −18.42 and −20.80 kcal/mol, respectively. Re-aromatization of the estrogen A ring is no doubt a strong driving force the loss of the C1-proton in addition to the reduction of the overall molecular dipole. These calculations only give the overall thermodynamic preference for the conversion of **1** to **2**, and while most proton transfers have small energy barriers, proton transfers to and from carbon atoms are known to occur at slower rates when compared to heteroatom proton transfers [20]. It is also noteworthy that the glycosidic bond is longer (weaker) in tautomeric form **1** when compared to tautomeric form **2**. These results still leave the question of when glycosidic bond cleavage occurs unclear. Since previous studies were able to measure the kinetics of the glycosidic bond cleavage (ΔG^‡^ = 26.8 kcal/mol) [16], we decided to model the key proton transfer step and compare the energy barrier with measured kinetic data.

**Table:** 

Compound	Relative Energy (kcal/mol)	N9-C1' Bond Length, (Glycosidic Bond) (Angstroms)	Dipole (Debyes)
T-2mα	0.00	1.4833	9.48
T-2mβ	0.54	1.4833	10.55
T-1mα	18.42 ^a^	1.4909	14.12
T-1mβ	21.34 ^b^	1.4927	13.76

a. ΔG T-1mβ *vs.* T-1mα = 2.92 kcal/mol b. ΔG T-1mβ *vs.* T-2mβ = 20.80 kcal/mol.

### 2.2. Kinetic Calculations of Proton Transfer from C1

**Figure 5:**
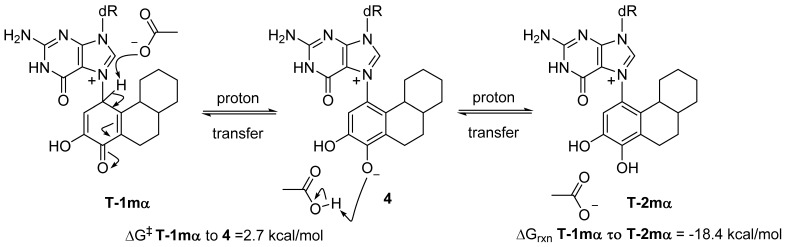
Modeling the kinetics of proton transfer at C1.

Most exergonic proton transfers have free energy barriers much less than the 26.8 kcal/mol measure previously for this reaction using UV/VIS spectroscopy. The two proton transfer steps that converts tautomer **1** to **2** are shown in Figure 5. Since proton transfers from carbon have higher energy barriers than proton transfers involving heteroatoms, we chose to model the transition state structure in going from **T-1mα** to phenolic form **4** (Figure 5). Scans of the potential energy surface are computationally more expensive than single geometry minimization calculations, thus we employed Hartree-Fock theory using the reduced MIDI! basis set of Truhlar and coworkers. Although smaller than the popular 6-31G(d) basis set, MIDI! has been shown to produce more accurate geometries [21]. The energies of the starting structure and transition state were then refined with a SPE calculation at the M06-2X/QZVP calculation as done previously (Figure 4). Both the geometry optimization and SPE were simulated in an aqueous environment.

The free energy barrier connecting **T-1mα** and phenolic form **4** was calculated at 2.7 kcal/mol. The saddle point of the transition state structure was confirmed by vibrational mode analysis that contained a single imaginary frequency (−1735 cm^−1^) that clearly shows the translation movement of the C1-proton towards the acetate anion. This small free energy barrier is consistent with a very exergonic proton transfer and is inconsistent with the experimentally observed kinetics of ribose loss, 26.8 kcal/mol [16].

### 2.3. Kinetic Calculations of Glycosidic Bond Cleavage

**Figure 6:**
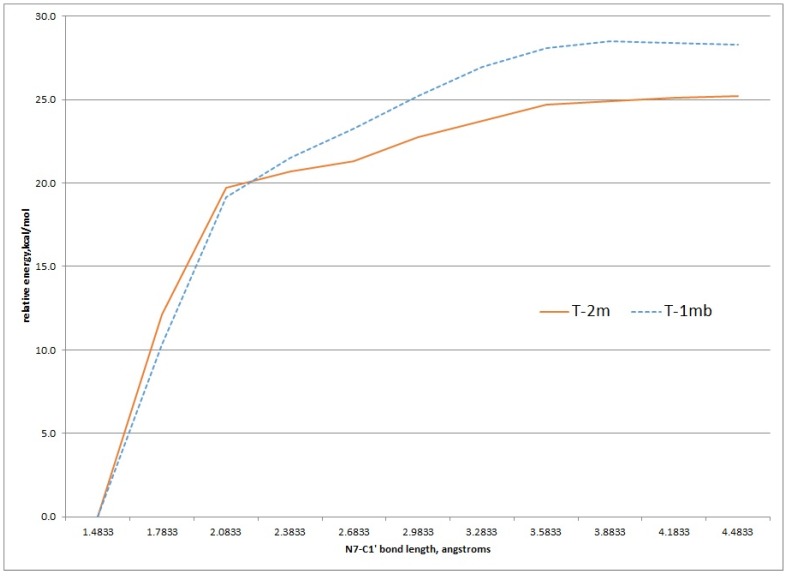
Relaxed potential energy scan of increasing N7-C1' bond length for the T-2m and T-1m tautomers, HF/MIDI! theory (PCM = water).

Using the structures of **T-2mα** and **T-1mα** as a starting geometries, a scan of the potential energy surface with respect to the N7-C1' bond was undertaken to see if the calculated value for this bond dissociation energy matched the experimental ΔG^‡^ of 26.8 kcal/mol for either tautomer. Figure 6 shows the results of scanning the N7-C1' bond length 3.0 angstroms in 0.3 angstrom increments. These relaxed scans were done at the HF/MIDI! level of theory modelled in an aqueous environment. The results show an increase of energy to just above 25 kcal/mol and 27 kcal/mol for the **T-2mα** and **T-1mα** tautomers, respectively. Both values are close to the experimental ΔG^‡^ value.

Transition state structures of unimolecular bond dissociation can be difficult to locate due to saddle points that are flat and not well defined and results are often compare the potential energy scan of the bond in question. We used the potential energy scans in Figure 6 as a starting point to locate transition state structures for both the **T-2mα** and **T-1mα** tautomers. Both calculation did converge on a structure possessing just one imaginary frequency but of low value indicating a flat saddle point maximum (−23 cm^−1^ and −19 cm^−1^ for **T-2mα** and **T-1mα**, respectively). We used these structures to calculate a free energy of activation for cleavage of the glycosidic bond (M06-2X/QZVP//HF/MIDI!, PCM = water). The resulting values of 26.2 kcal/mol for **T-2mα** and 25.5 kcal/mol for **T-1mα** are remarkably close to the experimental value of 26.8 kcal/mol. In order to distinguish between the two possible mechanistic paths (path A and B, Figure 2), we conducted isotopically labelled studies using a deuterium label at C1.

### 2.4. HPLC-MS Analysis of Deuterium Labeled Reaction Intermediate

**Figure 7:**
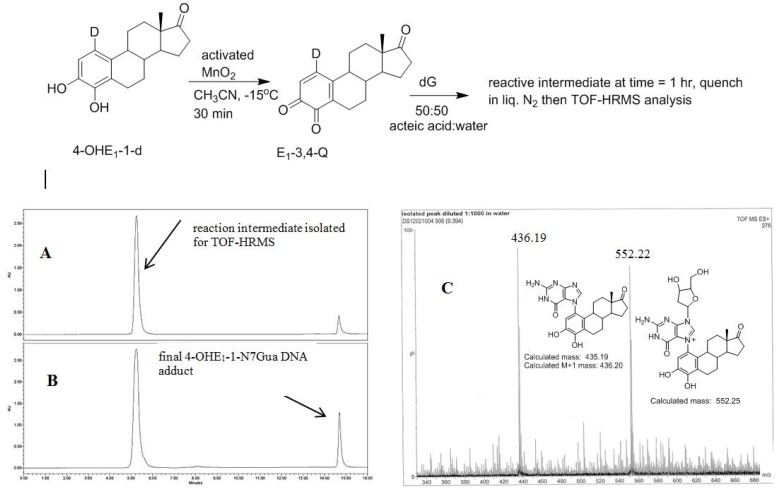
Oxidation of 4-OHE_1_-1-d to E_1_-3,4-Q-1-d and subsequent reaction with dG. (**A**) Reverse phase C-18 HPLC analysis after 1 h (B) Reverse phase C-18 HPLC analysis after 4 h (**C**) **T**OF-MS analysis of the early eluting peak in the positive ion mode.

The recent synthesis of 4-OHE_1_-1-d provides, after oxidation, an isotopically labelled form of E_1_-3,4-Q that would produce forms of **1** and **2** with different molecular mass. This allows identification of the reaction intermediate by MS. 4-OHE_1_-1-d was oxidized in acetonitrile at reduced temperature to form the E_1_-3,4-Q which was then added to a solution of excess dG in 1:1 acetic acid:water. Analysis of the reaction after 1 h shows an early eluting, polar compound (C18 reverse phase HPLC) that when isolated converts in a unimolecular fashion to the final 4-OHE_1_-1-N7Gua adduct (Figure 7). This early eluting compound was collected and frozen in liquid nitrogen until a time-of-flight high resolution mass spectrum (TOF-MS) could be performed to determine the mass of this intermediate.

A deuterium label at C1 allows for discrimination between tautomeric forms **1** (calc *m*/*z* of 553.25) which would contain the deuterium label and **2** (calc *m*/*z* of 552.25) which would have lost the deuterium label as a result of re-aromatization. Figure 7C show the TOF-MS of this reaction intermediate in the positive ion mode. The two prominent peaks observed in the spectrum have *m*/*z* of 552.22 and 436.19. *This data is consistent with structure **2** indicating cleavage of the glycosidic bond occurs after A ring re-aromatization (path B, Figure 2)*.

Since the reaction intermediate is positively charge, the peak at 552.22 *m*/*z* corresponds to parent peak, M, and the structure is the result of deuterium loss at C1 as a result of re-aromatization. The peak at 436.19 *m*/*z* is the fragment produced by loss of the ribose moiety (glycosidic bond cleavage). Since this fragment, which corresponds to the structure of the final 4-OHE_1_-1-N7Gua adduct, is uncharged, it is observed as the M + 1 peak when positive mode, electrospray ionization is employed. Both peaks at 552.22 and 436.19 *m*/*z* show a M + 1 peak that is slightly larger than the theoretical value of 30 and 25%, respectively. But the peak at 436.19 *m*/*z* corresponds to the known 4-OHE_1_-1-N7Gua adduct where the C1 proton is lost. So similar sized M + 1 peaks of both fragments is internally consistent with loss of a deuterium at C1.

To ensure deuterium loss does not occur during catechol oxidation, a sample of 4-OHE_1_-1-d (95% deuterium at C-1) was oxidized with activated MnO_2_ in acetonitrile to form E_1_-3,4-Q-1-d which was then reduced back to 4-OHE_1_-1-d with sodium borohydride. The resulting catechol was isolated and found to have retained greater than 90% of its original deuterium content via NMR analysis.

## 3. Experimental Section

### 3.1. Thermodynamic and Kinetic Calculations

Structures for **T-1-mα**, **T-1-mβ**, **T-2-mα** and **T-2-mβ** were constructed in ChemBio3D (PerkinElmer, Waltham, MA, USA) and initial geometries were generate by driving the dihedral about the C1(estrogen)-N7(guanine) and the N9(guanine)-C2' (ribose) bonds. The lowest energy conformer of both the α- and β-forms were then refined by MM2 molecular mechanics minimization and the resulting structures used as starting geometries for density functional calculations using Gaussian 09 software [22] (Gaussian, Wallingford, CT, USA). Geometries and thermal correction to the free energy were calculated using the M06-2X functional of Zhao and Truhlar using a 6-31G + (d,p) basis set. The effects of aqueous solvation were included in the geometry optimization by use of the polarizable continuum model (PCM) [23]. The resulting geometries were used to calculate the electronic energy at a higher level basis set of Weigend, QZVP [24], also using the M06-2X functional and PCM to model the effects of an aqueous solvent. The thermal corrections from the lower level geometry calculations were used to calculate the total free energy at the M06-2x/QVZP level of theory. The modeling nomenclature for this method is: M06-2X/QZVP//M06-2X/6-31 + G(d,p), PCM = water.

Modeling of the transition structure connecting **T-1-mα**, to 4 was initiated by performing a relaxed scan of proton C1 to the acetate oxygen in 0.1 angstrom increments at the HF/MIDI! level of theory. The highest energy structure on this path was used a guess for the transition state structure search using the synchronous transit-guided Quasi-Newton (STQN) method available in Gaussian09 (QST3 saddle point optimization) at the HF/MIDI! level of theory. The effects of aqueous solvation were included in the geometry optimizations by use of the polarizable continuum model (PCM). SPE of the starting structures and the transition state structure (verified by one imaginary frequency, –1735 cm^−1^) were calculated at the M06-2x/QVZP level of theory with the thermal corrections from the HF/MIDI! used to calculate the total free energy changes. The modeling nomenclature for this method is: M06-2X/QZVP//HF/MIDI!, PCM = water.

Transition state for glycosidic bond cleavage for **T-2mα** and **T-1mα** were conducted in a similar manner as above. The N7-C1' bond was scan in 0.3 angstrom increments and the results used to generate structures for STQN calculations.

### 3.2. Chemical and Materials

4-OHE_1_-1-d was made from estrone (Steraloids, Newprot RI, USA) as describe in a recent publication [17]. All other chemicals and solvents were purchased from Fisher Scientific Co. (Fair Lawn, NJ, USA) or Aldrich Chemical Co. (Milwaukee, WI, USA) and used as received.

### 3.3. Instrumentation

HPLC was conducted on a Waters 2690 Separations Module (Waters Corp. Milford, MA, USA) equipped with a Waters 2487 Dual λ Absorbance Detector using a methanol-0.1% aqueous acetic acid solvent gradient. TOF-HRMS of intermediate **2** was done on a Water Micromass Q-TOF Ultima API LC-MS. NMR analysis of deuterium content was obtained on a Bruker 400 MHz Avance III spectrometer (Bruker, Billerica, MA, USA).

### 3.4. Procedure for Generating Reactive Intermediate 2 and Determining Deuterium Stability during Estrogen Quinone Formation

4-OHE_1_-1-d (100 mg, 35 mmol) was dissolved in 5 mL of a 4:1 solvent mixture of acetonitrile:DMF, respectively. The solution was chilled to −15 °C and excess MnO_2_ was added and the suspension stirred rapidly for 30 min. The suspension was then filtered at −15 °C and split into two aliquots. One aliquot was added to a 5 fold excess of dG dissolved in 2 mL of an acetate buffer, 0.1 M pH 4.5. This mixture was brought to room temperature and HPLC samples analyzed at 1 and 4 h (Figure 6A,B) using a YMC, ODS-AQ C18 semipreparative column (5 μm, 120 Å, 250 mm × 10 mm, YMC America, Allentown, PA, USA). A gradient solvent system starting at 43% methanol and 57% water containing 0.5% acetic acid, at a flow rate of 3 mL/min was held for 5 min and then linearly increased to 100% methanol at 20 min. The intermediate **2** was collected as it eluted from the HPLC in a 4 mL amber vial and immediately immersed in liquid nitrogen. A sample of this intermediate was analyzed at the Nebraska Center for Mass Spectrometry (University of Nebraska-Lincoln) via HPLC/MS on a Water Micromass Q-TOF Ultima API LC-MS.

To the second aliquot of the estrogen-3,4-quinone was added 2.0 equivalents of methanolic NaBH_4_ at −15 °C. The solution was stirred for 5 min until all of the quinone’s orange/red color disappeared. Excess NaBH_4_ was then consumed by adding 0.5 mL of acetic acid at −15 °C. This reduction procedure is similar to that used by Pezzella [25]. Solvents were removed via rotovap and the crude redissolved in 1 mL of methanol and enough of the reduced 4-OHE_1_-1-d was collect for NMR analysis using the same HPLC conditions described above. NMR spectra shows an integration of the A-ring proton at C1 and C2 (C1 and C2 protons of 4-OHE_1_ have identical chemical shift) consistent with greater than 90% deuterium incorporation at C-1.

## 4. Conclusions

The reaction of estrogen quinones represents and interesting example of base excision from DNA by electrophilic binding to the N7-guanine nucleophilic site. While the details of depurination caused by simple alkylating agents has been well characterized [26,27], the quinone ring system leads to intermediate metabolites that makes the glycosidic bond cleavage event ambiguous due to the presence of different tautomeric forms. Our previous investigation into the *in vitro* reaction of E_1_-3,4-Q with dG established the following characteristic of estrogen adduction of dG: (1) the initial bond forming event is a proton assisted Michael addition of the N7-position to the *o*-quinone electrophile; (2) the reaction was stoichiometric with respect to the estrogen quinone; (3) a reactive intermediate is observed early in the reaction an coverts to final estrogen-DNA adduct in a unimolecular fashion [16].

What the prior work could not discern was which intermediate was present during glycosidic bond cleavage. Base on the UV/VIS spectroscopic we speculated that glycosidic bond cleavage might occur from the non-aromatic tautomer **1**. Using high level molecular modeling data in addition to isotopic HRMS data we can now establish the sequence of bond making/breaking that occurs when estrogen quinones form adducts with dG *in vitro*.

While these reactions were conducted in 50% acetic acid, the formation of intermediate **2** and the final product proceed in a similar fashion in acetate buffers up to pH 5.5; the *in vitro* reaction does require a proton source [16]. The proton environment *in vivo* does not reflect the conditions present here and the kinetics of tautomerization could differ *in vivo*. However, the thermodynamic trends presented should be similar. Also, the experimentally measured kinetic data of glycosidic bond cleavage was done from an intermediate isolated by HPLC (Figure 7A) which elutes in a solvent mixture of 43% methanol and 57% water containing 0.1% acetic acid, much less acidic than the reaction mixture. Calculations done using a polarized continuum of water are in good agreement with the measured kinetic data of glycosidic bond cleavage.

The importance of using the reaction of dG with E-3,4-Q in determining the structure involved during glycosidic bond cleavage is that analogous experiments cannot be done with the reaction of 2′-deoxyriboadenosine (dA) and E-3,4-Q. While E-3,4-Q does form an N3-adduct both *in vivo* and *in vitro*, the *in vitro* reaction only works with adenine not dA [28]. Apparently, the presence of the ribose ring sterically blocks access to the N3-position *in vitro* but not in the DNA. This means the early eluting intermediate seen in the HPLC (Figure 7A) cannot be examined with the adenine reaction since the ribose cannot be present at the start. This leaves only the dG reaction as a platform for mechanistic investigations.

**Figure 8:**
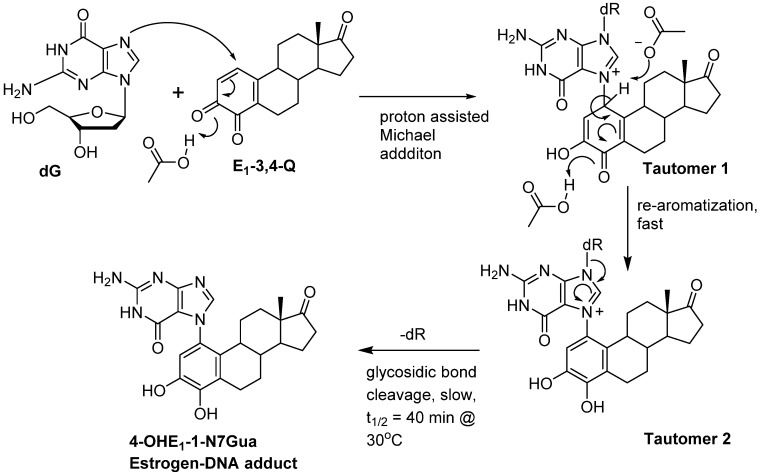
Revised mechanism of the reaction of E_1_-3,4-Q with dG.

Figure 8 shows the refined mechanism based on the results present in this article. The reaction starts with a 1,4-conjugate addition of the guanine nucleophile at the C1-poisition of E_1_-3,4-Q resulting in tautomeric form **1**. Tautomeric form **1** quickly loses the proton at C1 to form the aromatic A ring. This tautomerization/re-aromatization step has a strong thermodynamic driving force of *ca.* 20 kcal/mol with a small energy barrier of 2 kcal/mol and is essentially irreversible. The C1 proton loss is followed by a slower glycosidic bond cleavage. Future modeling studies designed to explore the difference in rates observed between guanine adducted by estrogen quinones *versus* adenine should use the isomeric form **2**.
